# Gender Differences in the Functional Limitations of Frail Older People Ageing in Place Alone in Italy

**DOI:** 10.3390/healthcare12222259

**Published:** 2024-11-13

**Authors:** Maria Gabriella Melchiorre, Marco Socci, Giovanni Lamura, Sabrina Quattrini

**Affiliations:** Centre for Socio-Economic Research on Ageing, IRCCS INRCA—National Institute of Health and Science on Ageing, Via Santa Margherita 5, 60124 Ancona, Italy; g.melchiorre@inrca.it (M.G.M.); g.lamura@inrca.it (G.L.); s.quattrini@inrca.it (S.Q.)

**Keywords:** ageing in place, frail older people, daily living activities, functional limitations, support, gender, mixed methods, Italy

## Abstract

Background/Objectives Older people with functional limitations find it difficult to age in place alone, without cohabiting with relatives. In light of this, this paper aimed to investigate possible gender differences in this respect among seniors living in Italy. Methods: The study presents findings from the IN-AGE (“Inclusive ageing in place”) study carried out in 2019 in this country assessing the ability of seniors aged 65 years and over to carry out basic and instrumental activities of daily living (ADLs and IADLs), in addition to two mobility limitations (going up/down the stairs and bending to pick up an object) and sensory limitations (hearing and eyesight). Qualitative/semi-structured interviews were administered to 120 older people living in three Italian regions (Lombardy, Marche, and Calabria). Quantitative and qualitative analyses were performed by differentiating between genders and among activities carried out autonomously, with help, or not performed (i.e., the senior is “not able”). Possible sources of support were also explored. Results: The main results revealed that cleaning the house, shopping, bathing/showering, and washing the laundry are particularly difficult, with men reporting greater difficulties than women. Moreover, for both genders, the family—especially children—represents the main source of help, in addition to public and private services, but the results differ between males and females. Conclusions: These results can offer insights for policymakers in the development of adequate gender-sensitive policies.

## 1. Introduction

Ageing in place, i.e., an approach allowing “older people to remain in their own homes for as long as possible”, reflects “a sense of attachment to home and neighbourhood, with improved wellbeing and social connectedness” [1] (p. 1), and enables seniors to stay integrated in their communities, thus avoiding or delaying possible institutionalisation [2]. The “home holds a lifetime of memories, offers an unparalleled sense of security and enables older people to feel in control of their lives” [3] (p. 4). Despite this positive value due to the benefits it has for older individuals’ quality of life, ageing alone at home without cohabiting relatives can also represent a crucial risk factor, leading to both negative health and social consequences, especially when functional and cognitive limitations in daily activities significantly impact the frailty of seniors and residential conditions do not meet their needs [3]. Overall, frailty involves demographic aspects, lifestyle, multimorbidity [4], and available social support, with social frailty representing a context of lacking/poor social resources that are necessary to meet the basic social needs of seniors [5,6]. In particular, living alone is a key indicator that affects social frailty, in addition to neighbourhood relations, social participation, and economic status [7].

According to a recent study by Ahonen and Kuivalainen [8], living alone differs in Europe by gender and country, with an overall proportion of 50% of women and 25% of men aged 75 and over living in a single-person household, with the female share being the lowest in southern countries (approximately 40%) and the highest in the Nordic ones (around 60%). Thus, following women’s longer life expectancy, it is more probable for them to live alone in later life, and, conversely, for older men to live, for instance, with their wives. In Italy, the shares of men and women aged 75 and over living alone are 20% and 50%, respectively. Data from ISTAT [9] indicate in particular that in 2020, almost half of Italian women aged 75 and over lived alone, with 29% living as a couple. The situation of men is reversed: 21.7% live alone and 68% live as a couple. More recent data from ISTAT [10] indicate that overall, 50% of seniors aged over 65 years lived alone in this country in 2023.

Another source regarding seniors aged 65 years and over with functional limitations reports that overall, in 2019 (the year of our survey) [11] in Italy, 46% of men and 57% of women had at least three chronic diseases. Furthermore, 9% of the former and 19% of the latter suffered from anxiety and depression. With specific regard to depression, the rate was 7% among men and 15% among women. Cases of senile dementia, including Alzheimer’s disease, were again lower among men (3%) and higher (5%) among women. Such a context of female disadvantage with regard to health in old age leads older women to use health services more frequently than men, especially specialist doctors. In addition to this, there is a lack of aid and assistance complained about mainly by women (70%), and difficulties in accessing health services with an associated renunciation of them for economic reasons (14% for men and 19% for women). The same source [11] reported that in Italy in 2019, around 11% (13.3% females vs. 7.5% males), had difficulty performing the basic activities of daily living (ADLs, e.g., personal care activities), 27% (35% females vs. 18% males) had difficulty undertaking instrumental activities (IADLs, e.g., domestic activities), and 28.4% (33% females vs. 23% males) had severe sensory (e.g., eyesight and hearing), cognitive, and mobility limitations. Older women are thus more limited than older men in all the activities mentioned above. According to EUROSTAT [12], in European countries (EU-27) in 2023, 52% of people aged 65 years and over reported long-standing limitations in their habitual activities due to health problems, with a confirmed gender health gap represented by 48.5% for males and 54.2% for females. In Italy, these values are lower: at 47.8%, 43%, and 51.5%, respectively. Overall, the available evidence on trends in the functional limitations of seniors in European countries indicates that older women suffer more than men from severe activity limitations [13]. In Italy, “women outperform men in terms of survival, while men have better health at older ages than women” [14] (p. 920).

When seniors age in place with functional limitations, care arrangements are crucial, as mentioned above, and differ among European countries, with informal/family care prevalent in southern countries and formal/institutional care prevalent in northern ones [15]. Italy is a traditional familistic country, with relatives still taking care of their seniors, among whom 50% receive support from them, whereas 17% receive it from paid workers (e.g., for housework and personal care) and 7% from friends/neighbours/volunteers [11,16]. Also, in Italy, older people with functional limitations are supported by the public sector, and related care interventions are mainly cash-for-care allowances that can be provided both at home and in residential facilities. The main monetary transfer is the National Disability Attendance Allowance (about EUR 500 per month), which is available to citizens certified as totally dependent. Moreover, but to a lesser extent and with a lower geographical coverage/intensity (i.e., a few hours a week) and limited available resources, frail seniors can apply for in-home services, e.g., the Integrated Home Care service (health and social) and the Home Care Service (social). Few seniors (only about 2%) live in residential care facilities. However, on the whole, Italian seniors prefer to live in their own home, and thus to age in place, rather than moving to a nursing home/residential facility [16]. Most older people in Italy indeed live in a home they own and almost a quarter benefit from some form of social rent, while only a minority live in homes with a rental contract on the free market. The title of ownership is strongly correlated with the economic conditions of older persons, and the majority of those who live in a home they own declare that they have no financial problems, especially men. It is worth highlighting the presence of significant architectural barriers inside the homes, e.g., the presence of stairs or steps which significantly limit the functional autonomy of frail and solitary seniors in carrying out some of the activities of daily life and in moving safely around their home environment. The propensity to implement renovations in their home environment often depends on the possibility of intervention and the ability to sustain the related expenses [17]. Another source [9] reports that 41% of the population aged 75 and over lives in a home with a private garden, and that 79% declare that their home has a terrace or balcony. However, 10% cannot count on an outdoor space, and 2% are in a condition of serious housing deprivation. These are seniors who live in a home with various problems (structural or brightness problems, or a house without a bathroom/shower), which can negatively impact their ability to carry out ADLs and IADLs. In old age, the presence of structural problems and/or architectural barriers, in addition to physical and cognitive limitations, might also increase the risk of falls and consequent possible injuries, especially for seniors aged 80 years and over. Such a context, in turn, is likely to lead to increased negative health outcomes for older people, e.g., reduced mobility and autonomy, disability, and hospitalisation, including the fear of falling again. All of this results in both societal and economic costs, with the crucial necessity to monitor and prevent such events in old age [18].

In light of the context depicted above with crucial implications for both genders, and considering the current ageing process, this study used findings from the “Inclusive Ageing in Place” (IN-AGE) research project [16] to explore the following: how frail male and female older people ageing in place alone in Italy carry out activities of daily living; the main difficulties they need to manage; and the support they receive in this regard. For this purpose, the following research questions were developed: (1) Which activities/functionalities are performed alone, with help, or are not performed at all due to inability by frail older people aged 65 years and over in Italy? (2) What are the main types of support they receive for carrying out the activities of daily living? (3) Are there gender differences with regard to both of the questions above? It is assumed that activities requiring mobility are overall the most difficult to perform, and that the gender dimension could impact the whole context. This follows, on the one hand, the greater physical limitations of older women in later life, as reported in the literature, but also considers the cultural aspects leading them to be more accustomed to carrying out, for instance, activities such as cleaning the house, cooking, washing the laundry, and arranging for the purchase of groceries.

The results of this analysis could provide useful insights for researchers and policymakers seeking dedicated care strategies for supporting frail seniors by taking into account their main difficulties and needs—which also depend on the gender dimension—thus facilitating the implementation of the most appropriate interventions while also considering the gender issue.

## 2. Materials and Methods

### 2.1. Study Design: Setting, Sample and Recruitment

The (mainly) qualitative IN-AGE survey was carried out in three Italian regions (Lombardy, North; Marche, Centre; and Calabria, South) from May to December 2019. It involved 120 older people (90 females and 30 males) who were recruited in one medium-sized urban site (24 units each, respectively, in Brescia, Ancona, and Reggio Calabria) and one rural/inner area (16 units each, respectively, in Oltrepò Pavese, Appennino Basso Pesarese e Anconetano, and Area Grecanica) in each region. To this end, the most degraded/peripheral urban districts [19] and inner areas, such as zones with increasing ageing processes and few health-social services [20], were selected. These three regions are considered representative of the different socio-economic development levels of Italy (higher in Lombardy; medium in Marche; lower in Calabria) [21].

A convenient purposive (non-probability) sampling method was applied, with included participants having characteristics that allowed for an appropriate exploration of the study topics and with no aim of statistical representativeness [22]. The inclusion criteria were as follows: men and women aged 65 years and over; living alone at home without cohabiting family members, or with a personal private care assistant (PCA) living in or helping on an hourly basis; intermediate mobility, between limited/reduced to within the home and outside the home with help; the absence of cognitive impairment (i.e., to be able to answer questions independently); and the absence of help from close family members (living in the same urban block/rural building) [16]. In our study, a simplified and non-holistic approach to frailty was thus adopted, viewing it as an overall condition linked to ageing and specifically to seniors aged 65 years and over who live alone without cohabiting relatives and have limited functional abilities. This primarily involves physical decline, which in turn implies reduced independent living and the need for support to perform both ADLs and IADLs, as indicated by some of the literature [23].

The recruitment of participants was carried out by the local sections of a major Italian association working with older people—i.e., Auser (Voluntary Association for Active Ageing)—operators of public home care/social services, and other voluntary associations (Anteas, Caritas). The respective territorial contacts/sites preliminarily checked the eligibility of seniors for involvement according to the inclusion criteria established for the study, especially with regard to their cognitive status and autonomy, on the basis of their own information and assessment. Recruitment channels provided, in particular, a preliminary screening to verify the cognitive capacity of participants based on the information they usually collect and assess with regard to users of public home care/social services and volunteering services. This information was further confirmed by the relatives of seniors participating in the survey. Thus, administrating a further cognitive test was not necessary. Then, the seniors were informed about the aim and procedure of the survey to attain their preliminary willingness to participate. The names and contact details of potential respondents (addresses and telephone numbers) were subsequently passed to the research teams, following initial verbal consent from seniors who agreed to be interviewed. Data saturation was set as a point where the recruitment of new participants and interviews provided redundant data and thus their inclusion did not add relevant information to patterns already collected [24], taking into account, however, the greater proportion of older women than men with functional limitations, as reported in the literature [11]. With respect to the participation rate, 58% of the seniors were ultimately included in the study (120 out of 208) whereas 42% declined, not only because of illness or hospitalisation during the survey, but also due to reconsidering about the interview despite an initial agreement.

### 2.2. Instruments, Data Collection, and Ethical Issues 

Semi-structured interviews were administered, including several closed/quantitative questions regarding socio-demographic characteristics, physical/functional limitations in daily living activities, and the main types of available social support (from people). Moreover, general qualitative questions (i.e., the dominant part of the study) explored more in depth their difficulties in daily activities and available support. It was decided to perform both quantitative research, to collect basic empirical information that can be synthetised by numbers numerically, and qualitative research, to explore the experiences and attitudes of participants, trying also to detect new meaningful insights into the studied topic using their own words. With particular regard to ADLs and IADLs, qualitative interviews help to better understand and interpret the difficulties in this respect beyond the simple and synthetic quantification of being able or not to perform them. Concerning the further intersection with the gender dimension, personal narratives allow for the highlighting of some nuanced differences and expressions/identities. Thus, the quantitative method helps in measuring the impact of gender bias on various aspects of social life, and the qualitative method helps in revealing the articulated processes “through which assumptions about gender and sexuality guide interactions, become embedded in institutions, and differentially affect life chances” [25] (p. 1).

The topics to be explored were generally addressed with questions adapted from previous studies [26,27] and from items contained in other research instruments to provide a preliminary conceptual framework with theoretically based definitions drawn from the literature, which allowed for the building of the questionnaire/topic guide. With regard to the quantitative aspects, physical/functional limitations were assessed regarding 12 ADLs-IADLs [28], both basic (necessary for survival) and instrumental (necessary to live independently within a community), integrated with two mobility limitations (going up/down the stairs and bending to pick up an object from the ground) and sensory limitations in hearing and eyesight [11,29,30]. The ADLs assessed include the following: getting into/out of bed, sitting/rising from a chair, dressing/undressing, washing hands and face, bathing or showering, and eating/cutting food. The IADLs assessed include the following: preparing food, shopping, cleaning the house, washing the laundry, taking medication at the right doses and at the right times, and managing finances. Sensory limitations were detected by means of the following questions: “Do you see well enough to recognise a friend on the other side of the street, or to read the label of a product, also considering the use of glasses and contact lenses?”; “Do you hear well enough to listen television at a volume that does not disturb other persons, considering the possible use of hearing aids?”. Regarding the overall limitations mentioned above, three levels of difficulty were considered: activity performed autonomously/alone, i.e., without the help of persons or aids; activity performed with help; and activity not performed, i.e., the senior is “not able” to carry out the activity (the latter is performed by others, or the senior is deaf/blind). Social support was addressed as a general closed question regarding the main types of assistance, by asking who helps seniors the most overall: relatives/children, friends, neighbours, operators from private services (e.g., Domestic Home Helper, DHH), those from public services (e.g., Home Care Worker, HCW), and PCAs. In addition to the quantitative aspects cited above, the following open question was administered: “Please, can you tell me more about the activities of your daily life you are able or not able to carry out more or less independently, with or without difficulty/help, and who are the people who help you the most?”. The question was generally proposed to allow interviewees to speak freely and share their experiences. However, the same question was proposed for each activity, or specifically for some activities in the case that the respondents were not talking so much in order to bring their attention to the topics of the survey and support their narratives.

Three psychologists and three sociologists (five females and one male) with extensive expertise in quantitative/qualitative data collection conducted the face-to-face interviews (two interviewers in each region). Despite their expertise, before the fieldwork they were invited to participate in a methodological training seminar that focused specifically on the aims and protocols of the study. Moreover, they performed a pilot test by conducting three test interviews (one in each region), which allowed both the appropriate refinement and improvement of the preliminary thematic framework. Each interview was conducted at the homes of seniors and lasted approximately 60–90 min. The interviewers audio-recorded and then transcribed the narratives in full/verbatim; in this task, they were aided by the use of a scheme that proposed the outline of the interview [16].

The Ethics Committee of the Polytechnic of Milan (POLIMI, Research Service, Educational Innovation Support Services Area) approved the study (authorisation n. 5/2019, 14 March 2019) before the data collection began. Also, a written informed consent form was signed by all participants, who were reassured of the confidentiality, privacy, and thus anonymity of their personal information. Moreover, codes to replace the sensitive information of respondents (i.e., name, address, and telephone number) were adopted. All this is in accordance with the ethical issues mentioned by the European General Data Protection Regulation (GDPR) n. 679 of 27 April 2016 [31].

### 2.3. Data Analysis

A mainly descriptive mixed-methods analysis of the 120 interviews was performed, without differentiating by regions and sites, to obtain an overall picture of the Italian context.

#### 2.3.1. Quantitative Analysis

The quantitative data, which were collected by means of closed questions (e.g., socio-demographic, activities performed alone/with help/not able, and type of support), were summarised with simple elaborations by gender using Microsoft Excel 2022 (Microsoft Corporation, Washington, DC, USA) and by calculating the related percentages (univariate and bivariate analyses). Regarding the level of physical limitations, four grades were considered, by gender: “mild” when an older person is able to perform all the activities (no activity labelled as “not able” is reported); “moderate” when an older person is unable to perform one or two activities; “high” when an older person is unable to perform three or four activities; “very high” when an older person is unable to perform five or more activities [32]. The results of the quantitative analysis are presented in tables below, with both absolute and percentage values (n. and %). In some cases, the sums of the latter do not correspond to 100% when individual figures have been rounded to simplify the reading. In other cases, the sums of the absolute and percentage values do not correspond to a total of 120 participants and 100%, such as when a single senior reported more responses for a single question. The first author, M.G.M., carried out the simple quantitative analyses which are presented in the tables.

#### 2.3.2. Qualitative Analysis

A qualitative analysis of open answers was carried out by following the five standard phases of the Framework Analysis Technique [33]: thoroughly/extensively reading the transcribed interviews (text format), naming macro/sub categories, identifying codes, building thematic charts to break down narratives (two-way matrices, with categories in columns and cases/interviewees in rows), and interpreting results [16,34]. The final codes were set according to their similarities and differences [35].

The overall qualitative analysis was carried out by nine researchers (three for each region, M.G.M., S.Q., and M.S. for the Marche region). In particular, for this paper, M.G.M. and S.Q. broke down 20 transcriptions each, in the charts. Then, M.G.M. and M.S. cross-analysed the charts, starting from the results of single sites (three urban cities and three rural/inner areas) and then merged the full dataset, thus detecting similarities and differences that emerged on the whole. Also, M.G.M. remained more focused on aspects such as difficulties in daily activities, while M.S. focused more on the gender dimension, according to their main expertise. The authors of the paper then discussed together the suitability, relevance, and correctness of labelling and interpretations of the contents.

A thematic qualitative content analysis was performed to identify the relationships among the themes that emerged [36]. This phase was carried out manually, without the use of dedicated software, as allowed by the literature [37,38], to conduct a thorough, line-by-line reading of the narratives and thus achieve a repeated exploration of the resulting charts and related thinking process, which is a typical aspect of qualitative analysis [22]. In this respect, the preliminary conceptual framework included in the topic guide facilitated this manual assessment. Software, when used, however, can represent a useful tool for storing and managing/organising the full data collection.

Narratives regarding activities (twelve ADLs and IADLs, two mobility limitations, and two sensory limitations) were categorised into five main groups (taking care of the house and laundry, taking care of themselves, moving within the house/building, moving outside the house/building and using money, and sensory conditions). One further group regards difficulties in some of the activities already mentioned (i.e., poor eyesight, hearing, and overall mobility), in addition to poor memory (but different from cognitive impairment, whose presence was fixed as an exclusion criterion), which can represent transversal functional limitations that hamper, in turn, other daily activities. Also, from narratives information emerged regarding who/what performs/helps with these activities. Compared with the closed quantitative question regarding support from others, which everyone answered, the response to the respective open question was not always specified, and a generic “I need someone who helps” was reported. Furthermore, seniors sometimes referred generically to care from their children without specifying whether they were sons or daughters. However, in some cases, additional people, such as grandchildren, brothers, and cousins, were added. Asking friends/neighbours for help “to not disturb” the family, and the use of aids as help for moving, e.g., a walking stick/crutch to go up/down the stairs, were also mentioned. Sub-categories were all coded by gender, and those regarding activities/functionalities, including transversal functional limitations, were also coded by type/level of difficulty (not able, with help, alone). This is apart from “overall poor mobility”, for which the overall level of functional limitation (mild, moderate, high, very high) replaced the more punctual level of difficulty mentioned above, since narratives in this respect were more general and not attributable to a specific daily activity. Moreover, when transversal functional limitations (apart from overall poor mobility) are assessed, the level of difficulty rating refers to the activity that is limited as a result, rather than the one that caused the limitation. The categories and coding that emerged and were applied are presented in Table 1.

The qualitative analysis of the findings has been integrated with/supported by the inclusion of several relevant verbatim quotations that emerged in the transcribed interviews [39]. For this purpose, each quotation was classified and coded with “IT” (for Italy) and a progressive interview number (1–120). For each quotation, the code also highlights both the gender of the respondent, i.e., “F” for female and “M” for male, and the type of difficulty of the mentioned activity, i.e., activity performed alone, with help, not able (e.g., IT-number_F/M_alone/with help/not able). The more general functional limitation level (mild, moderate, high, very high) is included in the codification, instead of the type of difficulty for the specific activity, when poor mobility is reported as a cross-cutting limitation, since the respective quotation regards an overall need for help as opposed to for a single activity (e.g., IT-number_F/M_mild/moderate/high). It must be highlighted that quotations are mainly from women due to the related larger female sample (90 vs. 30). Also, abbreviations are not reported within quotations so as to maintain the original words from the respondents. However, a small amount of editing has been carried out to improve the comprehension of excerpts without altering their meaning.

More detailed information (regarding the study design, setting, sample, recruitment, instruments, data collection, and data analysis) is available in a previous publication [16], from which the present Section 2 has been partly drawn and adapted.

## 3. Results

### 3.1. Sample Characteristics

The respondents were mainly people aged 80 years and over, with low (no diploma or primary school) and medium (middle school) educational levels, widowed, and living alone without a PCA. Regarding gender differences, women were more concentrated than men in the age group 80 years and over, whereas there were more men aged 85 and over; there were also more men than women in the younger age group of 65–74 years old. Moreover, women were less educated than men (lower presence among those with a middle or higher school education) and in great part were widows, with men emerging both as widows and as seniors who were divorced/separated. Men were also more concentrated than women among seniors living with a PCA (more women lived alone). Regarding mobility, an overall greater capacity to go outside the home, albeit with help, was found mainly for females. The monthly income was concentrated in the bracket EUR 601–1500 for both genders and was slightly more for men, whereas women were more present than men among those reporting a lower income, up to EUR 600 (Table 2).

More information on the sample is available in Melchiorre et al. [16].

### 3.2. Daily Activities, Difficulties, and Support: A Quantitative Picture by Gender

In this section, all daily life activities (ADLs and IADLs) and related difficulties, including those related to mobility and including sensory limitations regarding eyesight and hearing, are reported in detail. An examination of the level of physical and functional limitations precedes an in-depth analysis of the main activities carried out. An analysis of available support is also presented.

#### 3.2.1. Level of Functional Limitations

A total of 75% of older people report at least one activity they are not able to perform alone, and just under half of the sample have high/very high physical limitations. Moreover, men are more limited than women, with the latter presenting more cases in the mild/moderate group, and the former in the high/very high group (Table 3).

#### 3.2.2. Activities/Functionalities Performed Alone, with Help, and Not Able

When considering the whole sample, cleaning the house (56%), shopping (43%), bathing/showering (36%), and washing the laundry (34%) were reported as particularly difficult to perform alone/independently, and thus seniors reported mostly their total inability in this respect. This is in addition (but with lower values) to going up/down stairs, managing one’s finances, bending down to pick up an object, and preparing food (Table 4, first column).

The main actions that are mostly carried out with help (Table 4, second column) include going up/down stairs (71%) and bending down to pick up an object (58%), in addition to sitting/getting up from a chair, shopping, and getting into/getting out of bed. Furthermore, help is necessary for seniors reporting difficulties in seeing and hearing (40% and 44%, respectively). These cases require the use of aids (e.g., glasses and hearing aids). Among the main activities carried out independently/without help (Table 4, third column), washing hands/face (87%) and eating/cutting food (82%) prevail, followed by taking medications (63%) and preparing food (60%), whereas other activities are carried out “alone” by 46–55% of those interviewed.

When considering gendered experiences, the results indicate that women outperform men in almost all the activities carried out independently. Conversely, men have higher values than the women in almost all the activities not performed independently/not able. A more varied situation with regard to activities needing help emerged, e.g., men need more support than women do especially for sitting/rising from a chair (60% vs. 43%) and getting into/out of bed (47% vs. 40%), while women need more support than men do particularly for going up/down the stairs (76% vs. 57%), cleaning the house (43% vs. 13%), and washing the laundry (22% vs. 13%). Additionally, more women than men reported the need of help/aids for hearing (47% vs. 37%, respectively) (Table 5).

#### 3.2.3. Types of Available Support from Other Persons

Overall, greater support for older people with functional limitations in performing daily tasks is represented by family members (78%), particularly children (60%)—slightly more so by daughters (37%)—followed by private services (e.g., DHH), friends/neighbours, public services (e.g., HCW), and PCAs. With regard to gender differences, older men are supported more by both public services and PCAs than women are, with the latter being supported more by private services and friends/neighbours. Also, children help more female parents (Table 6).

### 3.3. Daily Activities, Difficulties, and Support: Quotations from Narratives by Gender

In this section, the quantitative results presented above are supported, enriched, and integrated with narratives and related quotations that emerged from the qualitative interviews carried out in the study. Moreover, when analysing groups of activities, types of help (from persons or aids) are considered. Quotations are presented in thematic groups of categories, according to Table 1, and in each group the activities/functionalities are listed according to the “not able” mode, i.e., starting from the one reported as the most difficult. More qualitative data/quotations are available in the Supplementary Material (File S1).

#### 3.3.1. Taking Care of the House and Laundry

Respondents’ narratives effectively depict how taking care of both the house and laundry is difficult overall, particularly for men and less so for women.

- Cleaning the House

Cleaning the house is the most difficult activity in old age, and it cannot be carried out any more by the majority of respondents, especially by men. Women, on the other hand, may still manage at least tasks with help, especially with regard to cleaning windows, shutters, and upper and lower parts. On the whole, help comes from HCWs, DHHs, PCAs, and relatives (as well as friends/neighbours in some cases).

*For cleaning I have a personal care assistant who used to take care of my wife when she was still alive. This assistant continues to visit once a week to take care of the house.*

(IT-89_M_not able)

*I cannot clean windows, shutters, and furniture, especially upper and lower parts. I have a domestic home helper who does these things.*

(IT-53_F_with help)

*My niece and my daughter sometimes help me with big cleaning jobs. For light ones, such as dusting, I do it by myself.*

(IT-117_F_with help)

However, when women clean their houses independently, some difficulties arise.

*I clean the house by myself, but very slowly. However, I do not clean as well as some years ago, when I was younger.*

(IT-70_F_alone)

- Washing the Laundry

Washing the laundry is no longer possible, particularly for men. Often, those who provide help for the house (PCAs, DHHs, HCWs, relatives), also do so for the laundry.

*I have the greatest difficulties with household tasks. A personal care assistant cleans my house and washes my laundry.*

(IT-104_M_not able)

*I have a domestic home helper who comes a couple of hours a week to clean and wash what is needed.*

(IT-96_M_not able)

*For washing clothes there is the home care worker.*

(IT-102_M_not able)

Women are better at washing, especially when they use a washing machine. However, sometimes women need help, either to start it or to hang out the laundry to dry. Help is therefore needed (from a DHH, relative, or friend/neighbour) for some steps.

*I use the washing machine sometimes. Other times my niece does it. I cannot hang out clothes because I have troubles with my arms.*

(IT-116_F_with help)

*I do the washing but I have some difficulty hanging out the laundry. I struggle with my arms! I get a neighbour-friend to help me for this.*

(IT-58_F_with help)

#### 3.3.2. Taking Care of Themselves

Taking care of themselves is confirmed overall as more difficult for men.

- Bathing/Showering

Having a bath or a shower alone in later life is truly problematic, especially for men. In this case, relatives, PCAs, and HCWs often provide help.

*My brother comes to shower me. I am not good on my feet and alone I risk falling!*

(IT-101_M_not able)

*A private care assistant gives me a shower.*

(IT-120_M_not able)

Women report more than men that they still the capacity to have a bath or a shower alone, although in some cases with some precaution, e.g., a little seat or the presence/supervision of a person.

*I still have a shower by myself, but I use a little seat. Because when standing I am afraid of falling.*

(IT-79_F_alone)

*I can shower alone but I prefer to have a person, for example a friend, close to me in case I need help, or something happens to me.*

(IT-6_F_alone)

However, both men and women sometimes need help to wash at least some parts of the body or to enter the bathtub.

*I struggle because I cannot wash by myself my back and feet.*

(IT-17_M_with help)

*Showering alone is a bit difficult. I cannot wash my shoulders and legs. A domestic home helper once a week helps me take a shower.*

(IT-34_F_with help)

*To take a bath I always need someone who helps me get into the bathtub, because my legs hurt and I cannot do it alone.*

(IT-107_M_with help)

- Preparing Food

With respect to preparing food, men are less autonomous and need help more than women, in collaboration/alternating with relatives, HCWs, and PCAs. Sometimes male seniors are indeed able to prepare (for dinner or for lunch) only “something simple”, e.g., pasta with sauce, a sandwich, or a cup of milk.

*Sometimes the home care worker prepares food for me. Other times I prepare by myself, for instance a simple pasta with sauce.*

(IT-101_M_with help)

*I have difficulty preparing food and thus I eat often some sandwiches that I am able to prepare on my own. Occasionally the home care worker prepares something.*

(IT-91_M_with help)

*The personal care assistant prepares some food for lunch, but in the evening, I take the milk and I make it by myself.*

(IT-88_M_with help)

Cooking simple food or making a sandwich is not, however, a male prerogative, because women too resort to such “strategies” even when they report needing little help.

*For eating I make do, today I had a sandwich for lunch.*

(IT-112_F_with help)

*For eating, I do something quick and simple.*

(IT-106_F_with help)

In some situations, both men and women refer to providing for themselves autonomously (mainly women), especially when cooking still represents a pleasant activity, a hobby, or a profession carried out in the past. However, non-demanding dishes are again mentioned as being prepared for dinner and lunch, while sometimes buying ready-made food represents an alternative.

*I take care of preparing food. I was a cook for several years.*

(IT-109_M_alone)

*I have always loved cooking. Modestly, I am also very good at it.*

(IT-10_F_alone)

*I make my own food, I get by. Sometimes I buy ready-made food and eat it at home.*

(IT-110_F_alone)

When seniors are absolutely unable to prepare food independently, especially males, there are family members or PCAs who provide food every day.

*My cousin comes to cook, always, and she does everything for me.*

(IT-113_M_not able)

*The personal care assistant prepares food for me.*

(IT-104_M_not able)

- Taking Medications

Again, men are less independent than women in taking medications at the right doses and at the right times. Thus, a daughter or a PCA is needed in this respect.

*My daughter puts the medicines in a box and then I know I have to take them. I could not take care of this on my own.*

(IT-107_M_not able)

*I have the list of medicines on the tables and the personal care assistant gives them to me when it is the right time.*

(IT-114_M_not able)

Those who are able to take medicine by themselves (especially women) use certain strategies, e.g., using the cell phone alarm clock, paying attention to the colour of the box of each drug, or writing the time and dosage on paper.

*I take several medicines, but I use the alarm clock on my mobile phone, which reminds me of the right time for each one.*

(IT-4_F_alone)

*I remember my medicines because I wrote their dosage and time on a piece of paper.*

(IT-101_M_alone)

- Dressing/Undressing

With regard to dressing/undressing, again males need (slightly) more help (from relatives and PCAs) than females do.

*My cousins and my son help me get dressed because I have circulation problems in my legs.*

(IT-113_M_with help)

*The private care assistant helps me get dressed and undressed.*

(IT-104_M_with help)

However, when women dress/undress alone, they need comfortable clothes.

*I dress and undress myself, but sometimes I struggle with uncomfortable clothes.*

(IT-79_F_alone)

When dressing/undressing alone is almost impossible, a PCA is the greatest “reference person” for both genders.

*Getting dressed and undressed by myself? No! The personal care assistant does this.*

(IT-30_M_not able)

*I need the personal care assistant for dressing! I cannot alone!*

(IT-95_F_not able)

- Eating/Cutting Food and Washing Hands/Face

The easiest self-care actions are eating/cutting food and washing one’s own hands/face, and these activities are almost “usual/common” for both genders. However, the main need for help in few cases (e.g., by a PCA) is for cutting the meat or for going to the bathroom, rather than simply eating or washing the hands and face.

*I cannot eat alone. My private care assistant cuts meat and fruit for me.*

(IT-120_M_not able)

*I can wash my hands and face, but someone has to take me to the bathroom.*

(IT-95_F_with_ help)

#### 3.3.3. Moving in the House/Building

Overall, greater female autonomy emerged.

- Going Up/Down the Stairs

Men are “not able” more than women to go up/down the stairs. However, women mention the need for some help in this respect much more than men do. The help is represented by a PCA or an aid, i.e., a walking stick or handrail. Additionally, more than one type of support is combined in some cases. Moreover, it is necessary to take one step at a time.

*I cannot go up and down the stairs. The personal care assistant helps me.*

(IT-94_F_with help)

*I climb the stairs with a stick, and hold on to the handrail.*

(IT-84_F_with help)

When men need help to go up/down the stairs, they also mention PCAs and handrails, in addition to children and crutches for walking.

*To go up and down the stairs I must always be accompanied by my children or the personal care assistant.*

(IT-104_M_with help)

*I go down the stairs with a crutch, and with the other hand I hold on to the handrail.*

(IT-120_M_with help)

In some cases, both women and men (more the latter) are not able at all to go up/down the stairs.

*I absolutely cannot walk up the stairs!*

(IT-101_M_not able)

*I cannot go down or up a flight of stairs, my back hurts. Absolutely no!*

(IT-10_F_not able)

- Getting into/out of Bed, Sitting/Rising from a Chair, and Bending

Men report the way, on the whole, getting into/out of bed, sitting/rising from a chair, and (slightly) bending to pick up an object are more difficult for them than for women. In these cases, PCAs usually help (totally o partly), but a stick can be useful too.

*The personal care assistant has to help me get out of bed because I have various pains when I move.*

(IT-114_M_with help)

*The private care assistant puts me on the chair. My legs cannot help me anymore!*

(IT-30_M_not able)

*To sit down and get up from a chair I need a stick.*

(IT-104_M_with help)

Women report more cases of autonomy in the three actions cited above and mention various “supporting strategies”, e.g., using a stick (this is not considered a help, however, as reported by a respondent above), being careful not to use a chair that is too low, or even looking for a grip to pick up an object that has fallen to the floor.

*I go to bed by myself with the help of a stick.*

(IT-84_F_alone)

*I can sit and get up from a chair as long as I do not sit too low because otherwise, I cannot get up.*

(IT-6_F_alone)

*To pick up an object from the floor, I hold on to a chair and I catch it.*

(IT-103_F_alone)

However, women also need help in some cases for sitting/rising from a chair, bending to pick up an object, and getting into/out of bed.

*Getting up from a chair by myself is problematic, I need help because my legs hurt.*

(IT-43_F_with help)

*Sometimes I go to bed alone, other times my personal care assistant helps me.*

(IT-103_F_with help)

Moreover, both genders reported difficulty bending for dressing socks and shoes. In these cases, relatives are mainly mentioned as help. In one case, a man often prefers to give up in this regard.

*My niece puts the socks on me, I cannot do it.*

(IT-116_F_not able)

*For me to put socks on and off requires a lot of effort. I often prefer not to wear them.*

(IT-56_M_not able)

Certain strategies are employed when seniors have difficulty bending over to wear socks and shoes. They report needing help, but are also able to accomplish it all by themselves when using certain precautions.

*To put on socks, I have to cross my legs but I can do it.*

(IT-17_M_with help)

*To tie my shoes, I put one foot on a chair and I can do it.*

(IT-89_M_with help)

#### 3.3.4. Moving Outside the House/Building and Using Money

Regarding both shopping and managing finances, respondents’ narratives highlight how women are more independent than men, especially for the latter activity. To emphasise this “still present” financial ability, an older woman reported she has been a financial manager for many years in the past.

*For the moment I am still able to go shopping alone.*

(IT-29_F_alone)

*I know how to manage my finances on my own, I was a financial manager for 40 years.*

(IT-4_F_alone)

- Shopping

However, both genders (mainly males) sometimes refer to not being able to go shopping, and in such cases they require having their shopping carried out and brought to them by family members or even neighbours. An old woman also resorts to a street vendor who passes right under her house.

*There is a very kind neighbour who helps me. He does my shopping and he also goes to get me medicines.*

(IT-14_M_not able)

*If I have to buy something I have my daughter or niece buy it for me. I cannot walk.*

(IT-94_F_not able)

*I go shopping from a street vendor, he has many things.*

(IT-73_F_not able)

Also, seniors (mainly women) need help (e.g., by a DHH or neighbour) to have at least the heaviest purchased goods carried home, or to reach the top shelves in the supermarket.

*I cannot carry heavy things. I try to do the shopping on the days when my domestic home helper comes, so she can bring them home for me.*

(IT-58_F_with help)

*I go shopping alone, even on foot, at the nearby supermarket, but I need someone to take things from the high shelves, I cannot!*

(IT-34_F_with help)

Moreover, both genders need help to be accompanied to the shop by children and grandchildren, or PCAs.

*I go shopping with my daughter.*

(IT-20_M_with help)

*To go shopping I can trust my niece. We go together.*

(IT-119_F_with help)

*If I have to buy something, the personal care assistant accompanies me.*

(IT-88_M_with help)

Another aspect that emerged regarding purchases is that help is needed for seniors especially to reach shopping centers outside the city where they live, because the historic “shops” that are available nearby charge higher prices.

*For shopping here there is a nice shop that has everything, but it is almost expensive. I buy most of the things at the supermarket which is a bit far from my house to save money. Luckily, there is always a friend who accompanies me there, to go and come back.*

(IT-75_F_with help)

Even when some nearby shops seem accessible on foot, seniors do not go out for fear of falling, especially if they need to reach the lower part of the city to do the shopping. Sometimes, it is very difficult to walk outside the house, especially when the road has several descents and ascents and nobody can provide support.

*The shops are all in the lower part of the city and I do not go there anymore. I am afraid of falling.*

(IT-70_F_with help)

*There are no flat areas here, everything is uphill and downhill! There are more distant flat areas where I could go shopping but I do not have anyone who can accompany me!*

(IT-112_F_not able)

- Managing Finances

Regarding finances, when seniors are not able (mainly men) or help is needed (also for moving), the latter comes from the children or from a sister, for both managing money (especially for important “affairs”) and making bank/post office withdrawals, or when there is a fear of reaching these places alone. Also, help is needed to manage the work contract with the PCA.

*I know how to manage my money, but I do not go to the bank. Also, if I have to make important decisions, I do not do anything if my children are not informed.*

(IT-89_M_with help)

*My sister goes to the bank. I am afraid to go there alone.*

(IT-53_F_with help)

*I know how much I have and how much I do not have, but my daughters help me: to take money from me, for payments, even for the employment contract with the personal care assistant.*

(IT-64_F_with help)

When help is needed only for going to the bank, the perception is different. One woman considers herself able alone, whereas another thinks that she needs help.

*My children accompany me to the bank but then I do all on my own, I know what I have to spend.*

(IT-79_F_alone)

*I have to go to the bank with some relative, but then I can do financial operations on my own.*

(IT-75_F_with help)

#### 3.3.5. Sensory Conditions: Eyesight and Hearing 

Overall, females report greater sensory limitations than males. Regarding eyesight, they are mainly not able or need aids. However, even with glasses, sometimes seeing is not easy.

*I do not see anything, I do not see people, I do not even see the clock!*

(IT-116_F_not able)

*I do not see very well. If things are written with big letters I can read, but if they are small, I cannot see them even with glasses.*

(IT-112_F_with help)

Females report greater difficulties for hearing in particular, and thus they need/use aids or turn up the volume of the television.

*I can hear the television because I have a hearing aid!*

(CAL_REG_G_19 F with help)

*When I watch television, I have to keep the volume high. Otherwise, I do not hear well and sometimes I do not understand the words.*

(IT-111_F_with help)

However, some seniors (mainly women) are not willing to use a hearing aid, a device to hear better is strictly rejected, or they have to give up such a purchase because it costs too much.

*I am a little deaf, I need a device but I do not want it.*

(IT-92_F_with help)

*I should buy the hearing aid, but it costs too much.*

(IT-62_F_not able)

Overall, men are more autonomous, both in eyesight and hearing.

*If I see a friend on the street, I recognise him and I can also read small labels.*

(IT-101_M_alone)

*I can hear television well and I do not need to keep the volume high.*

(IT-109_M_alone)

#### 3.3.6. Transversal Functional Limitations

Some limitations affect most/all activities of daily living (mainly reported by women), i.e., poor eyesight, poor hearing, poor memory, and overall poor mobility.

- Poor Eyesight

Regarding eyesight, difficulties in this respect are reflected in the inability to go shopping, prepare food, and manage one’s own finances. (activities thus delegated, e.g., to a HCW).

*A home care worker does the shopping for me, because I cannot see. I would also be able to cook, but owing to the maculopathy, I have difficulty preparing food. Sometimes I do not eat or I burn everything. Also, when I see myself here alone, I feel not hungry. Moreover, I cannot manage my money, and I have to ask someone for withdrawals from the post office, again since I cannot see!*

(IT-12_F_not able; for the three mentioned activities)

- Poor Hearing

Besides poor eyesight, poor hearing also significantly impacts daily life, such as for managing finances or going shopping.

*I do not go to the post office anymore. I cannot see or hear well.*

(IT-117_F_not able)

*I am struggling a bit with hearing. People have to raise their voices otherwise I do not understand. This is a great problem, especially when I have to go shopping or to the bank.*

(IT-6_F_with help; for both mentioned activities)

- Poor Memory

Poor memory greatly compromises some/all activities, e.g., shopping, managing finances, preparing food, and taking medications.

*When I go shopping, I make a list so I do not forget what I need to buy. I have some memory problems and this helps me!*

(IT-45_F_with help)

*My son helps me manage money and control all the expenses, because I have a bad memory.*

(IT-65_F_with help)

*I do not cook because I do not remember the ingredients to use!*

(IT-63_F_not able)

*To take medicine I am completely dependent on my caregiver because I do not remember them.*

(IT-3_F_not able)

- Poor Mobility

Overall, having poor mobility impacts all daily activities, even though this limitation is not explicitly referred to as a single activity by respondents. This negative occurrence is more commonly reported as a great general problem in old age, when some activities are impossible to perform (moderate to high level of functional limitations), and even when no activity—“not able”—is reported (mild level of functional limitations).

*Now that I have reached an old age, I no longer move like I used to, and this compromises everything in some way.*

(IT-57_F_mild)

*When I have big difficulties, I let it go. I do not do anything. Since my current mobility is truly poor.*

(IT-41_F_moderate)

*The lack of autonomy and mobility, owing to back problems, weighs on me. It weighs on me a lot!*

(IT-91_M_high)

#### 3.3.7. “To Not Disturb” the Family!

Notably, some seniors (mainly women) prefer not to rely too much on their children, or to ask them for support, since they do not want to disturb them, especially when they live in another city. Thus, sometimes, seniors turn to neighbours or friends for “small matters”.

*My daughter has her own job. She cannot always help me. To clean the house, I call a friend.*

(IT-50_F_not able)

*My son is very busy with his own family and work. I cannot disturb him for everything. I ask a friend for minor needs, for instance for shopping something.*

(IT-43_F_with help)

## 4. Discussion

**Figure 1:**
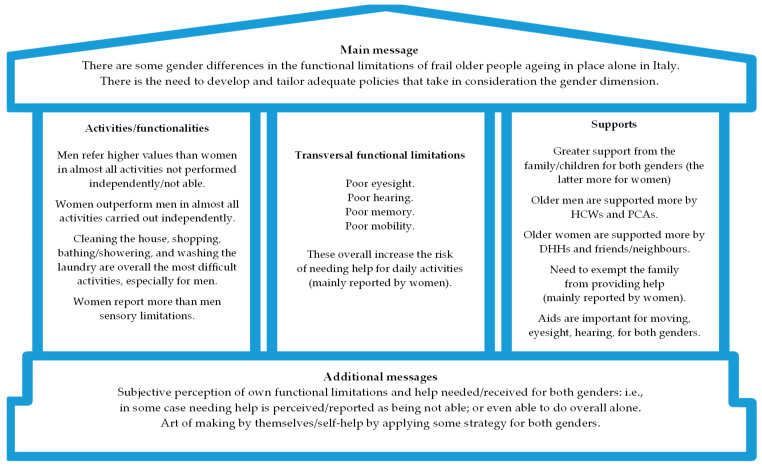
The Message House: framework for the contents which emerged from the study.

This study aimed to explore how frail older people of both genders carry out activities of daily living when ageing in place alone in Italy, including the difficulties and available support in this regard, with particular attention to possible gender differences. Overall, the findings indicate that women generally report lower values than men in almost all activities not performed independently, and vice versa with regard to almost all activities carried out alone/autonomously. Also, men report more high/very high functional limitations than women. The family, especially children, emerged as the greatest source of help for both genders. This general picture has, however, several gendered nuances, as discussed more thoroughly below. A synthesis of the framework of the main contents that emerged from the study, following the “Message House” method [40], is preliminarily presented in Figure 1. The aim of this figure is to summarise and organise the findings in a tool that facilitates their communication to the audience at a glance. Key components of the Message House method are the following: the core/main message as the central idea of the study (roof of the house); supporting messages as secondary points that reinforce/clarify the core message (columns of the house); and additional information that integrate the overall message (base of the house).

### 4.1. Daily Activities and Abilities vs. Difficulties: The Gender Dimension 

In our study, women perceived/reported themselves as more autonomous than men. In contrast, most of the literature generally reports different results, with females overall being more dependent and having higher rates of functional impairment than men [11]. This overall discrepancy can be attributed to the fact that in our study, a strict purposive sample was built, e.g., only seniors living alone or at most with a PCA, with intermediate mobility, and an absence of help by close family members. Moreover, in our sample, women are younger, have more cases of mild/moderate functional limitations, and are less supported by PCAs than men. Indeed, PCAs represent a form of paid help generally used more by older men with severe physical limitations. In our sample, women are more supported than men by children and friends/neighbours. These aspects could have impacted our overall results, which are however partly supported by some of the literature. According to Strauss et al. [41], women have greater difficulties in basic activities of daily living than men only at advanced ages, with gender differences in disability and morbidity thus becoming more evident among the elderly. Other authors [42] found that it is mainly older men with serious physical limitations who are supported by PCAs. A reason to explain why older men use more professional help is that it could also depend on their greater financial resources, as confirmed by data on pension benefits and beneficiaries of the Italian pension system as of December 2022 [43], according to which men receive 56% of the overall pension income, although women represent the majority share of all pensioners (52%). The average annual amount of income received by women is indeed lower than that of men by 27% (EUR 16,991 versus 23,167). Moreover, more men than women have a pension income above EUR 1500 per month (respectively, 58% vs. 38%), whereas the latter are often widows and have at most a survivor’s pension (and not their own work pension), which is usually of a lower amount since it is calculated as a percentage of the one due to the deceased husband. Chen et al. [44] reported that a wide social network (including friends/neighbours) was linked with lower disability for women. Similarly, Jiao et al. [45] indicated that social relationships may positively contrast with chronic conditions in female seniors. A study carried out in Norway with regard to adults aged 70 years or older [46] also indicated that independence in activities of daily living was associated with, among other factors, younger age and female gender. Moreover, ISTAT [11] highlights that 66% of seniors with functional limitations in Italy (65% females and 67% males), need help/support from persons or aids. Moreover, some authors [13,47] highlight how the lack of a partner in old age negatively impacts health, physical functionalities, and overall well-being, especially for men. Additionally, other authors [48] stress that women are generally more resilient than men, that is, are more capable of adapting and reacting to changes/limitations occurring in the life course, with biological, behavioural, and social factors also playing a role in this regard [49].

However, when some single/groups of activities are analysed, our findings present some more contact points with the wider literature on the topic, and some input also comes from cultural/gendered issues, as explained better below.

In particular, cleaning the house, shopping, bathing/showering, and washing the laundry were reported as difficult by both genders, although mainly by men. Overall, ISTAT [11] similarly reports that, aside from gender differences, 26% of persons aged 65 years and older in Italy are unable to perform heavy domestic activities (13% cannot perform light domestic ones), 15% are unable to go shopping, and 10% are unable to have a bath/shower alone. When these activities are included in the groups of categories mentioned in Table 1, they pertain mostly to taking care of the house and laundry, taking care of themselves, and moving outside the house/building. Taking care of the house and laundry indeed remain “typical” female tasks, with women thus being more dedicated and skilled in this respect, and also needing less support for these “typical” tasks in old age. Cerrato and Cifre [50] highlighted that gender is still an important dimension that impacts involvement in household tasks, with the traditional model indicating that “gendered” roles are still valid in some way. EIGE [51] also indicates that, in general (apart from age), housework tasks are highly gendered, with women performing more “feminine” tasks, such as cleaning, cooking, and laundry, whereas men perform more “masculine” tasks, such as home and garden care maintenance. Compared with men, women spend in particular much more time in doing housework every day, for at least one hour (81% vs. 20%). This cultural aspect could also be related to the greater attitude of women towards care activities, for both other people and themselves. Moreover, the cultural aspect could support the fact that men are less independent in going shopping, at least for purchasing groceries, with this in turn being partly linked to the preparation of food/cooking. Van Droogenbroeck and Van Hove [52] generally highlight how women are still the main people responsible for grocery shopping. Overall, cultural diversity can impact the link between gender and care [53].

When activities regarding moving in the house/building are considered, our results align more with the literature, with women being less able to go up or down stairs. ISTAT [11] indeed reports that when walking or going up or down stairs, older women show greater difficulties starting from the age of 65 (+3.4 percentage points compared to men), which raises to a gender gap of almost 20 points after the age of 85 (59% for women vs. 40% for men). The same source highlights that older women (14.5%) reported severe sensory limitations more than men (13%), in line with our findings in this regard. Moreover, self-care actions, such as eating/cutting food and washing one’s own hands/face, are easy for both genders in our study, as similarly reported again by ISTAT [11], which indicates how few seniors of either genders are unable to perform them (4–6%).

### 4.2. Transversal Functional Limitations Increasing the Risk of Needing Help

Our findings underscore the fact that some limitations, i.e., poor eyesight, poor hearing, poor memory, and overall poor mobility, can negatively affect several activities of daily living. Overall, excerpts from respondents’ narratives in this regard are reported mainly by women (who are also the majority in our sample), but they can be considered rather broad/unspecific, i.e., almost gender-neutral, and thus potentially applicable to frail seniors with functional limitations and, in turn, generally related to older age conditions. These limitations, when transversally considered, can indeed concern both genders.

The literature supports these findings, and indicates that older people with poor hearing and poor eyesight also generally report higher levels of functional limitations, particularly women [54]; such sensory limitations, especially when combined, increase the overall risk of negative health outcomes and shorter life expectancy [55]. Also, previous authors [56,57,58] have highlighted that cognitive impairment and consequent memory loss have crucial impacts on the ability to perform activities of daily living and on the general quality of life. Regarding Italy, ISTAT [11] indicates that especially older women aged 75 years and over (16%) reported greater difficulty with memory or concentration than men (9%). In particular, cognition requires “thinking, knowing, remembering, judging and problem-solving” [56] (p. 95), thus it is highly important for tasks such as shopping, managing finances, preparing food, and taking medications, as emerged in our study.

Poor mobility, which can significantly hamper daily activities, is generally reported by our respondents as a great problem in old age that it is not closely linked to a specific activity. Reduced mobility can indeed have an impact on almost all daily activities: cleaning the house (e.g., it is difficult to clean upper/lower parts); washing the laundry (difficult to hang out the laundry); having a bath/shower (e.g., help is needed for washing back and feet, or to enter the bathtub); dressing/undressing; moving inside (e.g., getting into/out of bed, sitting/rising from a chair) and outside (e.g., shopping and going to the bank/post office) the house. These activities require a certain overall agility, and some authors in this respect also found that mobility-related abilities in turn affect physical functionalities and overall health [59].

Regarding the mobility of seniors, another important result from our study is the need for help by respondents of both genders to be accompanied to the shops, especially when these are outside the city where they live or when seniors avoid going out for fear of falling (mainly women). Similarly, the need for help in going to the bank/post office emerged, even when there was a fear of reaching these sites alone. ISTAT [11] highlights that in Italy, mobility problems related in particular to difficulties in leaving the house, accessing buildings, or using private or public means of transport according to one’s wishes or needs are reported. Over four million older people (31.5%) have difficulty travelling for health reasons or functional limitations. Among older women, the share is close to 40% (22.3% for men). Moreover, from our narratives emerges the perception that difficulties in both going shopping and to the bank/post office are not as related to the management of purchases or of one’s own money/finances as they are to the need for help/accompaniment, or for being “replaced”, to go to the reference shops or to the offices responsible for processing some bureaucratic/administrative procedures (e.g., bank withdrawals). Thus, seniors feel that they still have the “head” but no longer the “legs” in this respect, and help especially from relatives is necessary. Several authors also stress that older people have diverse “safe mobility” needs, e.g., for shopping, attending medical appointments, and conducting business [60], and that concerns in this respect are also related to the fear of falling or of being the victim of a crime [61], all of which call into question both the physical and psychological risk factors for mobility limitations [62]. Regarding mobility, it is worth highlighting that in Italy, higher levels of seniors with mobility difficulties are recorded among those with sensory and cognitive limitations (74%), and this share exceeds 90% among those with serious limitations in personal care activities of daily living [11].

### 4.3. Who and What Supports Older Males and Females 

Overall, both people (relatives, DHHs, HCWs, PCAs, and friends/neighbours), and physical aids provide support. In particular, aids for moving, as well as for eyesight (e.g., glasses) and hearing, are reported by both genders. Concerning help from people, some gendered aspects did, however, emerge. Greater support indeed comes from the family, particularly from children for both genders, but more so for women. Also, older women are able to trust more than men in private services, e.g., DHHs, and friends/neighbours, and older men are supported more than women by both public services, e.g., HCWs, and PCAs. Respondents’ narratives depict well and confirm this picture, to which be added the aid of cousins, grandchildren, nieces, a brother, and also a generic “someone” in some cases.

Regarding overall help from people, ISTAT [11] supports these findings by stating that, on the whole, 85% of older people with serious difficulties in personal care report receiving help from family members (cohabiting or not). This percentage is 52% for those who benefit only from the help of family members, and 33% for those who are supported by family members together with other people (PCAs, HCWs, DHHs). Seniors who live alone compensate for the lack of support from cohabiting family members with greater recourse to paid help (44%) and in particular to the figure of the PCA (31%). Other authors report that when seniors have functional limitations, the family, when available, still represents the greatest source of help, particularly adult children, who can provide them both emotional and physical support [63]. Services, PCAs, and friends/neighbours are often complementary supports [64], with the latter assuming the role of primary caregiver when necessary [65]. Regarding neighbours, ISTAT [66] reports that relationships with them can be established and preserved over time, and thus neighbours represent persons on which one can count in case of need in everyday life owing to their proximity. Moreover, friends and neighbours often coincide in later life [16]. Notably, when our respondents are completely unable or need some help to manage their finances, only children are reported as the support (in one case a sister). Similarly, in a previous study [67], about 41% of seniors were found to be getting help with financial decisions, mainly from a spouse (when still alive) or from a child. In particular, when help comes from outside their household, this help comes mainly from a son, a daughter, or at least a professional financial advisor. Thus, difficulties in managing finances have consequences both for older people and their relatives [68], and psychological support in particular from family members in this respect represents an important pillar for seniors [69]. Managing money and other financial matters are indeed sensitive actions for older people, making trust in possible helpers, preferably children, necessary.

From a gender perspective, the literature highlights that in Italy, among older people who live alone and have children, 56.4% are used to seeing them daily; however, this is more true for women than men (60.5% vs. 43.4%) [9]. Other sources indicate that older women are more supported by DHHs than by PCAs, whereas older men are more supported by PCAs than by DHHs [42]. More generally, according to a meta-analysis by Tifferet [70] on gender differences in social networks, women give and receive greater social support, e.g., from family and friends, than men. Other authors [71] found that older men have higher levels of perceived material support, and that older women have more solid social ties. Also, a lesser amount of recent research has highlighted that older men receive more formal support than women [72]. Moreover, from our findings, the need to exempt the family from providing help, reported mainly by women, emerged. Seniors thus turn to friends or neighbours, when possible, in order not to disturb children too much—e.g., for “little matters”—especially when they live in another city. This particular result was also found in the previous literature [16]. In this regard, other authors [73,74] have indicated that, in some cases, older parents can experience feelings of guilt towards their children when they require their support, which in turn negatively impacts their overall well-being. Additionally, in our study, a slightly greater proportion of daughters, nieces, and female cousins who help were found, especially for cleaning the house, washing the laundry, and preparing food. This further gendered result is supported by the literature, which reports that more women than men provide help (38% vs. 33%) overall, and, moreover, that among the recipients of informal help, mothers are the particular demographic most found [66]. The only case of a brother mentioned in our narratives, who helps a male senior and provides him with a shower, also represents in some way a gendered-cultural issue, with some men probably feeling more comfortable with relatives of the same gender (when available) for personal care/hygiene.

Regarding support from aids/devices, few seniors in our narratives of either gender mention using them for mobility; generally, walking sticks and crutches, but also using a handrail for going up/down the stairs, are considered as “boosts”. According to a study by Martinelli et al. [75], very few (14%) older people are able to go out and carry out activities without the need for help, albeit with difficulty, and almost two-thirds (62%) go out, but they need help, i.e., instrumental aids such as sticks, walkers, wheelchairs, or human support. Other authors [76] reported that sticks were the most frequently used aids (72%), followed by a lower adoption of walkers (16%) and wheelchairs (7%). The fact that our findings report few people mentioning aids for mobility could depend (apart from the fact that we did not ask specifically whether seniors used them) on the usual negative association of this use with physical decline, thus leading to stigmatising attitudes and beliefs, as has also been reported by some authors [76]. Another study [77] similarly reported that some older people do accept neither their mobility loss nor the use of aids/devices in this regard, since they fear becoming dependent on them in their daily lives, with dependence having a negative connotation of being a burden for other people/relatives rather than a positive connotation of aid for their own personal, albeit “adjusted”, autonomy. It is also worth considering that mobility devices are different and require different minimal abilities to be used: sticks require sufficient balance, crutches require some body strength, and wheelchairs also require coordination. All these aspects could impact the refusal of mobility aids by seniors [78].

### 4.4. Cross-Reading of Some Statements

#### 4.4.1. Subjective Perception of Respondents’ Own Functional Limitations and Help Needed/Received 

From a cross-reading of some statements presented above, a different perception/reporting of self-limitations also emerged in certain cases, with regard to both genders. Some define themselves as incapable because they need help, while others define themselves as just needing help, and the line separating the two statements is very thin. For example, this double/reversed perception emerged when seniors needed help (from a PCA or DHH) for showering, dressing, eating, or sitting (women and men). Moreover, some (women) define themselves as “able alone” and others as needing help for managing finances, even though they report the same help of being accompanied to the bank by family members. Even using the stick to do something is differently perceived: for some it means being able to alone, while for others it implies needing help (women and men). Also, some authors generally report the existence of the “disability paradox”, suggesting that older people can consistently report a subjective well-being despite their functional limitations, and that this positive attitude is more prevalent in women than men [79]. In this regard, some socio-demographic and psychosocial aspects, e.g., economic status, self-esteem, and personality traits, can promote a reduced perception of one’s own limitations in daily activities and stimulate thus a positive reaction to functional decline [79]. Mitchell [80] also found that some people are capable of adapting to their own functional limitations so as to tailor their performance of daily activities accordingly, thus reaching a positive change in self-perception of their own overall physical/cognitive condition.

#### 4.4.2. The Art of Living by Themselves/Self-Help

Some adaptations to one’s own functional limitations, and in turn personal strategies to manage one’s own needs, further emerged from a cross-reading of some quotations described above. Even when the seniors in our study reported being able to perform daily activities on their own or needing/receiving a small amount of help, there was indeed still some effort involved, and thus with great tenacity some strategies were adopted by both genders. Following the art of living by themselves/self-help, some “escamotage” was applied. For instance, having a shower using a seat or with the supervision of a person, for fear of falling (women); preparing a cup of milk or a sandwich for dinner, and more generally cooking simple food or buying ready-made food (women and men); taking medicines with the reminder by a cell phone alarm clock or writing time and dosage on paper (women and men); wearing comfortable/large clothes (women); and crossing legs or putting one foot on a chair to wear socks and shoes (men). The literature has also addressed these issues by reporting that seniors often adopt strategies for managing daily activities [16]. Such strategies can be considered as enhancing one’s own talents, skills, and overall self-efficacy (i.e., belief in one’s own capacity to manage situations) and as practical approaches to coping with current and future needs for positive ageing and improving one’s own well-being [81]. Self-care practices can thus contribute to maintaining some functional independence [82].

### 4.5. Implications for Practices and Policies

In the wake of the main results of our study, some implications for policy makers can be provided, related to both genders and more generally for seniors with functional limitations.

From a gender perspective, our findings highlight that older men are less autonomous than women, especially for cleaning the house, washing the laundry, shopping, and bathing/showering, but also for preparing food, which are all usually activities performed by PCAs and HCWs in these cases. This implies the necessity for them to have support and guidance for accessing affordable and qualified PCAs, and for having a sufficient hourly amount of help from HCWs. In particular, the difficulties reported by older men in preparing food should be addressed by means of interventions, including nutrition-based strategies, to ensure they maintain proper nutritional and overall lifestyle patterns [49]. Women need more help for going up or down stairs (even though the share of females able to perform this task alone is greater than that of males), and thus the matter of aids is more urgent for them. In addition, supportive measures for children, mainly females, who take care of their respective elderly parents are needed, e.g., more adequate and personalised policies to reconcile work and care time. Affordable costs should also be associated with DHHs, who are more employed by women.

Regarding bathing/showering (more problematic for men) and sensory limitations (affecting more women), both genders could nonetheless benefit from interventions aiming to assure safety in personal care by means of adequate assessment tools [83] and relevant housing adaptations (e.g., grab bars in the bathroom, smart home sensors warning whether a door/window is closed or open). Such adaptations for ensuring the safety and security of housing could be extended to the overall removal of possible barriers hampering the mobility of seniors, including technological assets (e.g., remote assistance/healthcare), which are crucial in cases of emergency [3]. Additionally, interventions for reducing the barriers that limit access to the external environment should be implemented [13].

More generally, poor sensory limitations, mobility, and memory should be detected and monitored for all seniors by means of a ccomprehensive geriatric assessment carried out by a multidisciplinary team of social-health professionals [49]. In particular, policies to reduce or at least slow the incidence of such transversal criticalities, which in turn affect the performance of other daily activities, have the potential to increase the number of years free of functional limitations [55]. Also, the existence of a subjective perception of one’s own abilities, and the related need to “do it by themselves” in some circumstances, suggest the enhancement of the different residual functionalities and adaptation capacities for as long as possible [84]. This is particularly relevant with regard to the prescription, e.g., by medical doctors, of appropriate assistive devices/aids (sticks, crutches, walkers) for each senior, in addition to training on how to use them and avoid potential falls and injuries [78]. Moreover, from a general, not-gendered perspective, home care services should be implemented for supporting family caregivers when available and for supporting especially seniors who cannot receive help from their own relatives. This suggestion is made with the aim of maintaining as many older people as possible in their homes [46]. Another general issue concerning all older people living alone with reduced functional abilities, especially mobility, is the overall higher exposure to the risk of social isolation and loneliness [85]. In this respect, the integration of formal/informal, public/private, long-term care (LTC) home services could be effective overall [86], particularly for providing both social and psychological assistance to older people [87], with attention to interventions that are able to address the diverse functional limitations of males and females [88,89].

The implications described above are, in some way, summarised by the evidence-based recommendations of the “WHO Integrated Care for Older People (ICOPE) Guidelines” for assessing and managing declines in the intrinsic physical/mental capacity of all older people via community-level interventions. These should aim to improve their mobility, maintain their sensory capacities, prevent their cognitive decline and falls, promote their psychological well-being, and support their family caregivers [90]. Policymakers, health professionals, stakeholders, and caregivers could thus collaborate to implement adequate policies for all seniors, but with attention paid to a gendered approach [49] in adherence with the issues described above and responding to some of the gender differences in functional disabilities detected in our study. In particular, person-centred interventions that consider age, gender, and functional limitations as a whole, i.e., gender-sensitive and age-disability inclusive practices, should be developed [91].

### 4.6. Limitations

This study has some limitations to be considered. Seniors with cognitive impairments were not recruited with the aim of including only participants able to answer questions from the interview without help. Moreover, their cognitive status was assessed following self-reported information from the recruitment channels, without administering a specific cognitive test. The degree of functional limitations was self-reported; thus, it represents a proxy for seniors’ objective functional status [14]. A simplified, but limited, definition of frail older people is considered, i.e., persons aged 65 years and over, living alone, with functional limitations, and needing support for carrying out daily activities. Social support was not assessed by means of a validated scale, but only with ad hoc questions, and, moreover, the use of aids was not specifically assessed. Also, the level of functional limitations has been calculated only with regard to the number of activities that seniors are not able to perform by themselves. Regional and urban/rural comparisons are not carried out, mainly in order to present an overall national picture of the topic. The purposive sample built for the study does not allow for wide generalisation and statistical representativeness of the findings. It is also worth mentioning that some non-English papers and some Italian national statistics have been reported/discussed, since our study focused on the Italian context; this, however, reduces their readability by a wide international audience. Finally, caution needs to be used when interpreting percentage values in the tables, since the respective absolute values are sometimes very small.

Despite these limitations, the authors affirm the trustworthiness of the study, in particular of the qualitative analysis [92]. This was accomplished by performing a careful and accurate preliminary literature review and description of the study protocol, in particular with detailed notes regarding the data collection, transcription, and analysis processes. Moreover, the overall credibility and confirmability of the study were guaranteed by the following: several peer de-briefing sessions and close dialogue among researchers and interviewers with long-lasting expertise regarding the issue of ageing in place, as well as frequent dissemination seminars with several stakeholders and experts in the field to present and validate the findings collected step by step.

## 5. Conclusions

Our study revealed that, overall, older women were more independent than men in most daily activities. These findings are different from those in the previous literature, which describe women with higher rates of functional limitations than men. This, however, could partly depend on the purposive sample, which was built with particular restrictions—e.g., only seniors living alone or at most with a PCA, with intermediate mobility, and an absence of help by close family members. In addition, cleaning the house, shopping, bathing/showering, and washing the laundry were reported as particularly difficult, especially for men. The greatest source of aid for both genders is represented by the family, especially children, with the latter however being reported more by women. Further help is provided mainly by HCWs and PCAs for men, and DHHs and friends/neighbours for women. Deficits in mobility, sensory functionalities, and memory represent risk factors that cross-cut, and in turn affect, the other daily activities of both genders. Moreover, the subjective perception of one’s own limitations and the need to provide for themselves, at the least by adopting some strategies, were reported by both males and females.

These results can offer useful insights for researchers and policymakers for the design and implementation of supportive interventions aimed at relieving frail seniors with the functional limitations appropriate to those ageing in place alone, and their caregivers. This is true both generally and when considering the gender dimension in particular. Future research could thus analyse more in-depth the issue of ageing in place, focusing on gendered experiences and with regard to different contexts (e.g., community and care homes). Moreover, more research is needed to explore and compare the issue of difficulties and strategies to overcome functional limitations in later life with regard to seniors living in couples/with elderly partners or with children and grandchildren, including the aspect of possible intergenerational support.

## Figures and Tables

**Table 1:** Categories and coding.

Macro-Categories	Sub-Categories	Codes
Taking Care of the House and Laundry	Cleaning the house	Activities are: performed alone/in autonomy (without help) performed with help (of persons; of aids for moving, e.g., stick/crutch) not performed (respondents are not able/activities performed by others) Gender of respondents: male, female
	Washing the laundry	
Taking Care of Themselves	Washing hands and face	
	Bathing or showering	
	Dressing/undressing	
	Preparing food	
	Eating/cutting food	
	Taking medication	
Moving in the house/building	Getting into/out of bed	
	Sitting/rising from a chair	
	Going up/down the stairs	
	Bending to pick up an object	
Moving outside the house/building and using money	Shopping	
	Managing finances	
Sensory conditions	Eyesight	Functionalities are: performed alone/in autonomy (without aids) performed with help (of aids, e.g., glasses) not performed (respondents are not able/almost deaf or blind) Gender of respondents: male, female
	Hearing	
Transversal functional limitations	Poor eyesight	Impacted/compromised activities are: performed alone/in autonomy (without help) performed with help (of persons) not performed (respondents are not able/activities performed by others) Gender of respondents: male, female
	Poor hearing	
	Poor memory	
	Overall poor mobility	Level of functional limitations: mild (no activities “not able”) moderate (one-two “not able”) high (three-four “not able”) very high (five or more “not able”) Gender of respondents: male, female
Support from persons	Type for each activity (when reported)	Family (e.g., children/daughter grandchildren/niece, brother, cousin) Public services (e.g., home care worker, HCW) Private services (e.g., domestic home helper, DHH) Personal/private care assistant (PCA) Friends/neighbours (also “to not disturb” the family) “Someone” Gender of respondents: male, female

**Table 2:** Sample characteristics by gender (n = absolute values).

Characteristics	Female		Male		Total	
	n	%	n	%	n	%
Age Groups (years)						
67–74	10	11	7	23	17	14
75–79	15	17	4	13	19	16
80–84	24	27	4	13	28	23
85 and over	41	46	15	50	56	47
Education						
No diploma	11	12	3	10	14	12
Primary school (5 years)	42	47	13	43	55	46
Middle school (3 years)	13	14	7	23	20	17
High school (3–5 years)	21	23	7	23	28	23
University/similar (3–5 years)	3	3	-	-	3	2
Marital Status						
Single	11	12	5	17	16	13
Divorced/separated ^1^	6	7	10	33	16	13
Widowed	73	81	15	50	88	73
Living Situation						
Alone	72	80	21	70	93	78
With PCA	18	20	9	30	27	22
Mobility						
Only/Mainly in the home ^2^	34	38	14	47	48	40
Also outside the home with help ^3^	56	62	16	53	72	60
Monthly income brackets (EUR)						
Up to 600	10	11	2	7	10	8
601–1500	66	73	23	77	89	74
1501–2500	13	14	4	13	17	14
Over 2500	1	1	1	3	2	2
Total respondents	90	100	30	100	120	100

^1^ This includes two male respondents still married but not cohabiting with their spouses (de facto separated); ^2^ This also includes respondents able to move outside the home very rarely, i.e., less than two times a week and only if accompanied or with aids (canes, walkers); ^3^ Respondents are able to move within the home and also outside at least two times a week, only if accompanied or with aids (canes, walkers).

**Table 3:** Level of physical/functional limitations by gender (n = absolute values).

Level ^1^	Female		Male		Total	
	n	%	n	%	n	%
Mild	26	29	4	13	30	25
Moderate	27	30	6	20	33	28
High	19	21	8	27	27	22
Very high	18	20	12	40	30	25
Total respondents	90	100	30	100	120	100

^1^ The level of physical/functional limitations is based on 12 Basic and Instrumental Activities of Daily Living (ADLs and IADLs), two mobility limitations (going up/down the stairs and bending to pick up an object), plus sensory limitations in hearing and eyesight. Mild = no activities “not able”, Moderate = one-two, High = three-four, Very high = five or more.

**Table 4:** Activities/functionalities performed alone, with help, and not able (n = absolute values).

Activities ^1^	Not Able		With Help		Alone	
	n	%	n	%	n	%
Cleaning the house	67	56	43	36	10	8
Shopping	52	43	56	47	12	10
Bathing or showering	43	36	44	37	33	28
Washing the laundry	41	34	24	20	55	46
Going up/down the stairs ^2^	28	23	85	71	7	6
Managing finances	25	21	40	33	55	46
Bending to pick up an object ^2^	24	20	70	58	26	22
Preparing food	23	19	25	21	72	60
Taking medication	17	14	27	23	76	63
Eyesight ^3^	16	13	48	40	56	47
Dressing/undressing	7	6	47	39	66	55
Hearing ^3^	7	6	53	44	60	50
Getting into/out of bed	5	4	50	42	65	54
Sitting/rising from a chair	3	3	57	48	60	50
Eating/cutting food	3	3	19	16	98	82
Washing hands and face ^4^	1	1	15	13	104	87
Total respondents ^5^	120	100	120	100	120	100

^1^ Activities are sorted according to the “not able” mode (decreasing values). They include 12 Basic and Instrumental Activities of Daily Living (ADLs and IADLs), two mobility limitations (going up/down the stairs and bending to pick up an object), plus sensory limitations in hearing and eyesight; ^2^ Mobility limitations; ^3^ Sensory limitations; ^4^ Only a person who is not able to wash hands and face due to severe and several physical limitations; ^5^ In all cases, more activities are mentioned (the sums of absolute and percentage values are higher, respectively, than 120 and 100%).

**Table 5:** Activities/functionalities performed alone/with help and not able, by gender (n = absolute values).

Activities ^1^	Not Able					With Help					Alone				
	Female		Male		Total	Female		Male		Total	Female		Male		Total
	n	%	n	%	n	n	%	n	%	n	n	%	n	%	n
Cleaning the house	44	49	23	77	67	39	43	4	13	43	7	8	3	10	10
Shopping	37	41	15	50	52	42	47	14	47	56	11	12	1	3	12
Bathing or showering	30	33	13	43	43	33	37	11	37	44	27	30	6	20	33
Washing the laundry	22	24	19	63	41	20	22	4	13	24	48	53	7	23	55
Going up/down the stairs ^2^	16	18	12	40	28	68	76	17	57	85	6	7	1	3	7
Managing finances	15	17	10	33	25	31	34	9	30	40	44	49	11	37	55
Bending to pick up an object ^2^	17	19	7	23	24	52	58	18	60	70	21	23	5	17	26
Preparing food	14	16	9	30	23	18	20	7	23	25	58	64	14	47	72
Taking medication	9	10	8	27	17	20	22	7	23	27	61	68	15	50	76
Eyesight ^3^	13	14	3	10	16	36	40	12	40	48	41	46	15	50	56
Dressing/undressing	4	4	3	10	7	35	39	12	40	47	51	57	15	50	66
Hearing ^3^	5	6	2	7	7	42	47	11	37	53	43	48	17	57	60
Getting into/out of bed	2	2	3	10	5	36	40	14	47	50	52	58	13	43	65
Sitting/rising from a chair	2	2	1	3	3	39	43	18	60	57	49	54	11	37	60
Eating/cutting food	1	1	2	7	3	15	17	4	13	19	74	82	24	80	98
Washing hands and face ^4^	1	1	-	-	1	12	13	3	10	15	77	86	27	90	104
Total respondents ^5^	90	100	30	100	120	90	100	30	100	120	90	100	30	100	120

^1^ Activities are listed according to the “not able” mode (decreasing values). They include 12 Basic and Instrumental Activities of Daily Living (ADLs and IADLs), two mobility limitations (going up/down the stairs and bending to pick up an object), plus sensory limitations in hearing and eyesight; ^2^ Mobility limitations; ^3^ Sensory limitations; ^4^ Only a person who is not able to wash hands and face due to severe and several physical limitations; ^5^ In all cases, more activities are mentioned (the sums of absolute and percentage values are higher, respectively, than 30, 90, 120, and 100%).

**Table 6:** Types of supports/resources by gender (at least one type of help; n = absolute values) ^1^.

Types of Support ^2^	Female		Male		Total	
	n	%	n	%	n	%
Family	71	79	23	77	94	78
Children ^3^	55	61	16	53	71	60
Daughters	33	37	11	37	44	37
Sons	32	36	8	27	40	33
Friends/neighbours	39	43	11	37	50	42
Private services	43	48	7	23	50	42
DHH	36	40	8	27	44	37
Public services	31	34	12	40	43	36
HCW	19	21	9	30	28	23
PCA	18	20	9	30	27	23
Total respondents	90	100	30	100	120	100

^1^ The values in the table do not concern the number of family members, friends, etc. who help, but rather the number of older persons who reported at least one help of the respective type (one case with family helping = more than one family member helping); ^2^ In some cases, more types of help/support are possible (the sums of absolute and percentage values are higher, respectively, than 30, 90, 120, and 100%); ^3^ Both sons and daughters in some cases.

## Data Availability

All relevant data supporting the findings (i.e., absolute values, quotations) are within the manuscript. Additionally, a minimum dataset with quantitative data presented in this study are openly available in Mendeley at https://doi.org/10.17632/m4jrt9snhv.1 (accessed on 20 September 2024). The charts with all original verbatim transcriptions (in Italian) are not publicly available due to privacy/ethical restrictions, that is to their containing information that could compromise the privacy/anonymity of research participants. (e.g., include names of persons and locations and other potential identifiers of respondents).

## Supplementary Materials

The following supporting information can be downloaded at: https://www.mdpi.com/article/10.3390/healthcare12222259/s1, File S1, Additional qualitative data/quotations.
