# Supplementary material for: Gender Differences in the Functional Limitations of Frail Older People Ageing in Place Alone in Italy
Source: Healthcare (Basel). 2024 Nov 13;12(22):2259. doi: 10.3390/healthcare12222259 (PMC11594082; doi:10.3390/healthcare12222259)
Supplement: Supplementary file 1 [file healthcare-12-02259-s001.zip › healthcare-3268638-supplementary.pdf]

Supplementary Material - File S1

Additional qualitative data/quotations (according to sub-sections which are presented in par. 3.3. of the manuscript).

3.3. Daily Activities, Difficulties, and Support: Quotations from Narratives by Gender

3.3.1. Taking Care of the House and Laundry

<u>Cleaning the House</u>

I cannot clean my house, a home care worker sent by the municipal social service takes care of it. **(***IT-20_M_not able)*
A cousin of mine comes for cleaning, she does everything needed. (IT-113_M_not able)
I have a neighbour/friend who lives in my same building. She helps me for something, for instance for cleaning a little the house. (IT-86_F_with help)

<u>Washing the Laundry</u>

My cousin washes the laundry. (IT-113_M_not able)
The washing machine does the laundry, I load it. I do also the hanging out. (IT-97_F_alone)
I manage the washing machine and then I hang out all. I can also iron something. (IT-98_F_alone)
For the laundry I have the washing machine and then I hang out the clothes. Moreover, I still wash some little things by hand as well. (IT-111_F_alone)
I wash with the washing machine, but the domestic home helper starts it, since I am not able to do it. I also hang out but only sometimes. (IT-115_F_with help)

3.3.2. Taking Care of Themselves

<u>Bathing/Showering</u>

To take a shower I need someone, for instance the home care worker. (IT-88_M_not able)
The domestic home helper helps me get safely in the bathtub and take a shower twice a week. (IT-15_F_with help)

<u>Preparing Food</u>

Preparing food is something I love, a hobby. (IT-14_M_alone)
I still cook on my own, I still do it well. (IT-42_F_alone)
I cook for myself, sure, but I do not prepare elaborate dishes. (IT-41_F_alone)
I sometimes prepare something to eat by myself, while other times my daughter brings me cooked food. (IT-107_M_with help)
My niece prepares food for me every day and brings it home. (IT-102_M_not able)

<u>Taking Medications</u>

I recognise my drugs from the box. I cannot be wrong in this way. (IT-12_F_alone)

<u>Dressing/Undressing</u>

I dress by myself a little but the personal care assistant prepares my clothes so that I have everything at hand. (IT-88_M_with help)
I get dressed and undressed alone, it takes me some time but I can manage this with comfortable, loose and simple clothes. (IT-78_F_alone)

<u>Eating/Cutting Food and Washing Hands/Face</u>

If I have to eat meat, I need help. I have the private care assistant cut it for me. (IT-95_F_with_ help)

3.3.3. Moving in the House/Building

<u>Going Up/Down the Stairs</u>

When I go up the stairs, I take the handrail, and also, I have the stick. I go slowly, I take one step at a time. (IT-53_F_with help)

<u>Getting into/out of Bed, Sitting/Rising from a Chair, and Bending</u>

If something falls on the floor, I cannot bend down to pick it up. Someone has to provide this for me. (IT-107_M_not able)
If I have to bend down or kneel, then I have great difficulty getting up without help. (IT-82_F_with help)

I cannot put on socks and shoes because I cannot bend over them. (IT-43_F_not able)
I use elastic stockings and my son puts them on for me. (IT-113_M_with help)
I dress by myself but I need help tying my shoes. (IT-67_F_with help)

3.3.4. Moving Outside the House/Building and Using Money

I manage my money. I know how to do the math, I pay bills, and I go to the post office. (IT-111_F_alone)

<u>Shopping</u>

For purchases I have to ask my daughter who lives nearby and she brings to me what I need. (IT-107_M_not able)
I trust neighbour if I need something. I ask her to bring to me the heaviest things such as water bottles. (IT-36_F_with help)
I go always shopping with my children. (IT-101_M_with help)
I go shopping with my son on Saturday. (IT-9_F_with help)
My personal care assistant always accompanies me to buy what I need. (IT-64_F_with help)

<u>Managing Finances</u>

My son manages the money, he does everything. He goes to the bank to withdraw my pension. (IT-113_M_not able)
The money is managed by my daughter who has the authority to withdraw money from the post office. (IT-117_F_not able)

I manage my finances, but I have problems signing up some related documents. My hand is shaking and the bank refuses my electronic signature because the device does not recognise my current shaking signature. (IT-17_M_with help)

3.3.5. Sensory Conditions: Eyesight and Hearing

From afar I do not recognise the people. I cannot read a package label if it is not big enough, even with glasses. (IT-115_F_not able)
I cannot see well and I have to depend on others to dial a telephone number or to read a private document. Anyway, I do not like letting others read my documents! (IT-12_F_not able)
I can see quite well even from afar. I only wear sunglasses. (IT-109_M_alone)

I can see clearly because I had cataract surgery. Medical doctors only recommended that I use sunglasses. (IT-89_M_alone)
I still see well because I had a cataract surgery, first one eye and then the other. The medical doctor told me not to use glasses and I was fine with this. (IT-119_F_alone)
I have trouble hearing. I am planning to buy a hearing aid. (IT-82_F_with help)
Many times, my children tell me to get hearing aids, but I do not want them. I put the television closer and what I understand is enough. (IT-89_M_with help)
I can hear television well and I do not need to keep the volume high. (IT-109_M_alone)

3.3.6. Transversal Functional Limitations

Poor Eyesight

The personal care assistant does the shopping because I cannot see anymore. (IT-3_F_not able)

Poor Memory

I take care of medicines by myself, so far, I have an "iron" memory and I hope it does not abandon me! (IT-83_F_alone)

If I meet persons on the street, I do not recognise them. This does not depend so much on the eyesight, because I see well, but rather on lapses in my memory. (IT-98_F_with help)

Poor Mobility

I realise I cannot do things anymore as I would like. When I go out, I have slips when walking, and all this stops me. (IT-50_F_high)

3.3.7. "To not disturb" the family!

I try to do everything alone so as not to disturb anyone. Of course, if my leg hurts, I ask my children for help in case I need to take a bath. (IT-86_F_with help)
